# Pakistani healthcare professionals’ perceptions of communication with patients and their relatives about hereditary breast cancer: a qualitative study in a LMIC

**DOI:** 10.1007/s12687-023-00639-x

**Published:** 2023-02-23

**Authors:** Shenaz Ahmed, Hussain Jafri, Wajeeha Naseer Ahmed, Andleeb Khanam, Yasmin Rashid, Mushtaq Ahmed

**Affiliations:** 1 Leeds Institute of Health Sciences, School of Medicine, Worsley Building, University of Leeds, Leeds, LS2 9NL UK; 2 Fatima Jinnah Medical University, Lahore, Pakistan; 3 Thalassaemia Society Pakistan, Lahore, Pakistan; 4 Health Department Punjab, Lahore, Pakistan; 5 Yorkshire Regional Genetics Service, Leeds, UK

**Keywords:** Public hospital, Limited resources, Hereditary, Breast cancer, Pakistan, Relatives, Communication

## Abstract

Pakistan has the highest incidence and mortality rates of breast cancer in Asia, with high numbers of patients diagnosed at a young age suggesting the possibility of an inherited cancer syndrome. Communication of hereditary breast cancer (HBC) risk information with patients could enable earlier detection of the condition in relatives and reduce mortality rates. This study aimed to explore perceptions of healthcare professionals (HCPs) in Pakistan about communication with patients and their relatives about HBC. Semi-structured qualitative interviews were conducted with eighteen HCPs during March to May 2020 in Lahore. Thematic analysis shows the HCPs were generally supportive of informing patients themselves about HBC, but believed it was the patients’ role to inform their relatives. HCPs also highlighted important barriers to communication with patients about HBC, including (i) patients’ low socioeconomic status and educational attainment; (ii) high prevalence of the social stigma of breast cancer; and (iii) lack of health resources and facilities to provide genetic testing for HBC. In conclusion, HCPs would value the development of interventions to support communication between HCPs and patients. They also highlighted the need for interventions to support intrafamilial communication about HBC. Much research and political support are needed to address patient, social, and systemic-level barriers to facilitate communication about HBC.

## Introduction

Pakistan has the highest incidence and mortality rates of breast cancer (BC) in Asia (GLOBOCAN 2018; Hanif et al. 2009). It is the most commonly diagnosed cancer among women in Pakistan and is a leading cause of death (Sarwar and Saqib 2017). In 2018, BC constituted approximately 37% of newly diagnosed cancer cases in women in Pakistan, affecting more than 34,000 women and resulting in over 17,000 deaths (GLOBOCAN 2018). These figures are based on small proportions of the population, so exact figures are unknown in Pakistan due to the lack of a national cancer registry (Badar et al. 2020). Nevertheless, trend analyses suggest that incidence and mortality rates of BC are increasing; therefore, it is a major public health burden in Pakistan (Zaheer et al. 2019).

A substantial number of BC patients in Pakistan are diagnosed at a young age. For example, a study on the age and stage of breast cancer in a breast unit in Karachi, Pakistan, with data collected from 1994 to 2016 (10,018 patients), shows 22% of patients were diagnosed at 31–40 years old (Soomro et al. 2018). In Pakistan, BC is more common in women in their 20s compared to the rest of the world (Mamoon et al. 2009). This may be attributable to family environment, lifestyle factors, or genetic inheritance, or a combination of these. Generally, diagnosis of BC at 40 years or younger tends to imply genetic susceptibility, with an increased probability of carrying a pathogenic variant in a breast cancer gene like BRCA1/2 (NICE 2013). Research suggests awareness of the condition, and use of surveillance methods can increase the chances of early detection of BC in low–middle-income countries (LMICs) (Anastasi and Lusher 2019; Naz et al. 2016), and that women with a relative with BC are likely to uptake surveillance services for BC (Cook et al. 2009). Consequently, healthcare professionals (HCPs) have a duty of care to discuss hereditary breast cancer (HBC) risk information with patients and their families to enable early detection of the condition (NICE 2013). In Pakistan, genetic testing for BC genes is limited; therefore, in this context, HBC refers to a family history of BC and/or early onset of BC.

Research in high-income countries (HICs) shows that communication of cancer risk information is complex and difficult (Akanuwe et al. 2020), and there is a lack of evaluated interventions to support HCPs’ communication about HBC risk (Starkings et al. 2020). In addition, such interventions tend to focus on supporting intrafamilial communication about genetic test results (Lieberman et al. 2018). Such interventions are not suitable for supporting communication between HCPs and patients in LMICs, including Pakistan, where testing for HBC is only available via private healthcare services. Furthermore, interventions developed in HICs rarely prioritise accessibility for individuals with low levels of literacy or take into account reasons for delayed healthcare-seeking behaviours in LMICs, such as perceptions of the financial burden of cancer treatment, reliance on spiritual healing/herbal medicines, perceived stigma of BC, and social discrimination (Mamoon et al. 2009; Banning and Hafeez 2009; Bottorff et al. 2007; Khan et al. 2015). Communication of HBC is further complicated for HCPs in Pakistan by suboptimal or missing patient pathways from primary to secondary services, reliance on patient initiative to seek secondary healthcare, and financial constraints (Tovey and Broom 2007; Hameed Khaliq et al. 2019; Hameed Khaliq et al. 2018).

Overall, there is a need to develop interventions to facilitate HCPs’ communication with patients suspected of having HBC (family history of BC and/or early onset of BC) in Pakistan and their at-risk relatives to facilitate early detection of the condition. To support the development of such interventions, the aim of this study was to explore perceptions of HCPs’ in Pakistan about communication with patients and their relatives about HBC.

## Method

### Study design

The study employed a qualitative approach, with semi-structured interviews. The interview guide was developed to explore participants’ experiences and perceptions of the prevalence of HBC and whether or not it is an issue in Pakistan, communication with patients about HBC, communication with relatives about HBC, and barriers and facilitators to such communication.

### Participants

Purposive sampling was used to recruit HCPs involved in the healthcare of patients with BC. Medical and nursing staff working in the oncology, radiology, pathology, and surgery departments of a public hospital in Lahore were approached by their respective heads of department. Details of interested participants were passed on to the researcher who further explained the study and arranged interviews with consenting participants (written informed consent for publication was obtained).

### Data collection

The interviews were conducted over a 3-month period (March–May 2020), at the participants’ workplace. Interviews lasted approximately 45 min. All interviews were conducted in Urdu, audio recorded, and translated and transcribed into English by a bi-lingual researcher.

### Data analysis

Reflexive thematic analysis was used to guide data analysis (Braun and Clarke 2006; Braun et al. 2018), underpinned by the ‘Normalisation Process Theory’, a framework for developing, evaluating, and implementing complex interventions. This approach is compatible with applied health research and can enable the exploration of participants’ subjective experiences (Campbell et al. 2021). The analysis involved six phases (see Table 1), using deductive and inductive approaches (Braun and Clarke 2006). Initial data analysis involved classifying and organising data using subheadings based on participants’ responses to the interview questions, where the resulting themes were similar to the interview guide (deductive analysis using NVivo 12, Sage Publications). Following a further review of the transcripts, themes were added, modified, merged, and changed iteratively (inductive analysis). Researchers’ subjectivity is an important analytic resource in reflexive thematic analysis (Braun and Clarke 2020). Therefore, SA analysed all the transcripts as an experienced qualitative researcher with expertise on the research topic. Also, SA is of Pakistani origin and based in the UK, so researchers based in Pakistan reviewed SA’s interpretation of the data to ensure the inclusion of any cultural nuances from their perspective. During analysis, differences by gender, department, and seniority of the HCPs were explored but not found.

**Table 1:** Process of coding and thematic analysis

Phase 1: familiarisation with data—WNA translated and transcribed the data in English. SA read and re-read the transcripts in English and listened to the interview audio recordings in Urdu wherever clarification was needed.	
Phase 2: generating initial codes—SA generated codes based on questions in the interview guide, and patterns of meaning beyond the scope of the interview guide.	
Phase 3: searching for themes—SA and WNA initially categorised codes according to the topics in the interview guide (deductive analysis).	
Phase 4: reviewing potential themes—SA reviewed, added, modified, merged and changed these initial themes as analysis progressed (inductive analysis), to better understand HCPs’ perception of communication about HBC with patients and their relatives.	
Phase 5: defining and naming themes—SA, WNA and HJ discussed, refined and agreed the titles and interpretations of the themes. This phase enabled the inclusion of these researchers’ subjectivity as “a resource for knowledge production” rather than a credibility and validity assessment, allowing a more nuanced understanding of the data in this international collaborative study.	
Phase 6^a^: producing the report—SA produced the first draft of the report, with the support of HJ and MA’s clinical expertise to draft the “Discussion”. All the authors contributed to reviewing and revising the manuscript.	

^a^Analysis involved moving back and forth between the phases

## Results

Interviews were conducted with 18 HCPs. See Table 2 for participants’ demographic characteristics. The qualitative findings are presented below with anonymously attributed illustrative quotes.

**Table 2:** Demographic characteristics of the participants

Gender	Male	1
	Female	17
Age (years)	Mean (range)	38.4 (25–55)
Role	Nurse	4
	Consultant	6
	Registrar	5
	House officer (equivalent to UK foundation years doctor)	3
Department	Breast clinic	3
	Oncology	7
	General surgery	5
	Pathology	3

### Perceptions of communication with patients about HBC

HCPs agreed it was their responsibility to inform patients suspected of having HBC about the implications of the condition for their wider family:

…this responsibility falls totally on our shoulders... It’s important to tell patients that members of your family need screening as they are also at risk. (HCP12)

Whilst HCPs acknowledge this responsibility, they highlighted barriers to communication about HBC at patient, social, and systemic levels, and suggested solutions to facilitate such communication.

### Patient-level barriers and facilitators to communication: well-being, socioeconomic status, and education

HCPs explained that patients’ mental and physical well-being was an important factor in considering communication about HBC. They believed the diagnosis and treatment of BC were physically and emotionally exhausting for patients; therefore, patients were unlikely to be interested in information about their relatives’ risks of HBC:

Patients won’t tell others because they are going through such a difficult process themselves… For her, she herself is the most important person, rather than hearing about others… (HCP3)

Nevertheless, HCPs suggested timely approaches to discussing HBC could be implemented. For example, after meeting patients’ needs and developing rapport with them:

…managing their anxiety and stress is more important than genetic counselling. But if the patient is admitted, we get to know them, then genetic counselling is okay. (HCP9)

HCPs also clarified that patients attending public hospitals were usually from lower socio-economic backgrounds and had little or no education; therefore, communication about HBC would be challenging. Patients were believed to generally lack basic understandings of BC, knowledge about their family history of cancer, and as having misconceptions about BC as a communicable disease:

...patients that come to us aren’t educated enough. ...explaining genes to them can be difficult. (HCP16)

…they lack this knowledge (BC history). (HCP15)

They think this might be communicable. They ask ‘could I eat with the family?’ (HCP2)

Hence, HCPs believed patients were unlikely to understand key concepts necessary to recognise the implication of HBC for relatives. Therefore, HCPs suggested the development of information resources to support communication with patients about HBC, in written and audio formats to ensure accessibility:

…we should have something written to give to patients. (HCP18)

…most people here cannot read… So, material should be mostly verbal… we need to use technology. (HCP3)

### Social-level barriers and facilitators to communication: stigma of breast cancer

Based on clinical experiences, HCPs described the social stigma of BC for patients as a barrier to communication about HBC. They understood patients were often blamed for having BC, hence feared being shamed by others, social isolation, abandonment, and divorce:

The majority of patients don’t disclose it to their family members... they are accused ‘this is all because of your bad deeds’. (HCP2)

…people avoid patients and families with a diagnosed patient. (HCP11)

...their husbands divorced them straight away, saying ‘she has cancer’. (HCP10)

HCPs suggested the stigma of being diagnosed with BC also led to asymptomatic women/relatives avoiding screening:

…women fear gossip when people get to know they went for breast screening, because there must be something wrong. (HCP5)

HCPs were concerned about the adverse social implications of advising patients to discuss HBC with asymptomatic relatives. They believed stigma attached to BC led to patients’ reluctance to disclose their diagnosis and relatives’ reluctance to consider screening. Therefore, HCPs emphasised the importance of addressing the stigma of BC through public awareness campaigns, including the use of social media platforms and discussion by religious scholars to reach diverse populations:

…awareness in the masses is the only way…to help the general public understand the benefits of early detection. (HCP16)

Everyone’s got a mobile phone. Everyone’s a pro at using Facebook, TikTok …so it would be very easy to spread information. (HCP9)

…there’s a need to include religious scholars… in the villages… to educate our women. (HCP2)

HCPs also believed the Government should raise awareness about BC, and develop and provide public BC screening programmes, with equitable access in remote, low-resourced areas:

…the Government should aim to reach people in remote areas. (HCP7)

### Systemic-level barriers and facilitators to communication: resources and health facilities

HCPs described various systemic barriers to communication with patients about HBC. They highlighted the lack of guidance documents and assessment tools to identify patients with a family history of BC.

...we need assessment procedures and proper tools to calculate the risk of relatives. (HCP9)

They also had concerns about the lack of genetic testing to conclusively identify families at risk of HBC because the condition may not necessarily be inherited:

…we cannot be sure it’s inherited, because we don't have the facility to identify it as genetically inherited. (HCP2)

Furthermore, HCPs believed communication about HBC should include advice for patients and relatives about genetic testing for BRCA genes. However, they seemed conflicted about giving such advice because genetic testing was only available at a high cost in private hospitals, and most of their patients struggled with the financial implications of their treatment. The lack of realistic options for genetic testing for patients attending public hospitals was perceived by HCPs as another barrier to communication with patients about HBC:

...our tertiary care hospitals don’t have BRCA testing... patients have to go privately (HCP10)

…they can’t afford it (genetic testing). They don’t even have money for their own treatment and medication, or to even travel to hospital... so no one will spend on tests for someone who’s ‘at-risk’. (HCP14)

Therefore, HCPs suggested the need for government interventions and financial support to facilitate the development of resources and facilities to support communication about HBC:

We have a lot of financial challenges and there’s no government support. (HCP9)

### Perceptions of communication with patients’ relatives about HBC

HCPs recognised the importance of informing patients’ relatives about HBC. They believed that understandings of the risks of developing BC, recognition of its signs and symptoms, and vigilance could enable relatives to seek earlier diagnosis and treatment. HCPs recognised that patients had lay understandings of inheritance because they were often concerned for their relatives, particularly daughters:

Patients have an understanding that daughters may be susceptible to breast cancer. (HCP12)

However, HCPs also believed most patients and relatives were from disadvantaged groups and had low levels of education, so would find it challenging to understand HBC. Together with limited time and resources, HCPs clarified that informing patients’ relatives about the implications of HBC themselves was beyond their remit and that their focus was on the patient. Instead, they advocated the use of trained genetic counsellors, who could dedicate more time than doctors to enable patient and their relatives to better understand HBC:

You cannot expect quality when the number of patients outweighs the number of doctors… We should have genetic counsellors… to guide patients about it (HBC). …we can’t do it effectively due to time constraints. (HCP17)

HCPs generally believed the responsibility to inform relatives was the patients’ and their family physicians’ (as a reliable and credible source of information) and highlighted the need to develop written information to support intrafamilial communication:

...patients should tell at-risk relatives about it. (HCP11)

What a layman says has minimal effect... the most important person is the family physician. (HCP4)

…some leaflet along with verbal communication would be best. …even if there’s one person in the family, he can read it to relatives. (HCP15)

HCPs recognised that written information alone may not be sufficient to enable information dissemination about HBC within families. This was because patients may be apprehensive about informing relatives, and/or relatives may not be receptive to information due to the stigma of BC:

…we can use a leaflet, but patients may not pass information on to their relatives. (HCP7)

…most (families) aren’t receptive… (HCP16)

### Perceptions of HBC as an issue

There was variation in HCPs’ perceptions of the prevalence of HBC in their patient population, hence the extent to which they believed it was an issue. Some HCPs quoted “almost 30% of patients” based on their experiences and others quoted “5–10%” based on the literature. Although, the latter also questioned the extent to which these figures were applicable to their patient populations given differences in the demographics of their patients (particularly age) and those in the literature:

…research articles state that breast cancer is a disease which is usually diagnosed in old age, but the age range of most patients with breast cancer here is 28-35 years. (HCP9)

HCPs also had concerns about the extent to which the incidence of BC within a family could be considered as “genetically inherited” versus “familial due to lifestyle choices”:

...genetic inheritance is a predisposing factor, but the precipitating factors are in our social environment… smoking and alcohol in females is common nowadays. (HCP4)

HCPs added that although a patient’s family history may indicate HBC, confirmatory genetic testing was necessary to ascertain a clinical diagnosis; otherwise, there was a risk of mislabelling families with HBC:

Unless we test patients for mutations, we can’t claim that it’s inherited. (HCP2)

Overall, based on their observations of large proportions of young women with BC and multiple women from the same family with the condition, HCPs acknowledged the importance of communication with patients about HBC. However, they also cautioned against labelling families as having HBC without a genetic diagnosis.

## Discussion

HCPs acknowledged their responsibility in informing patients about HBC and the implications of the condition for family members, but then it was the patients’ responsibility to inform their relatives. Although there was a consensus that HCPs should support intrafamilial communication, HCPs also suggested the need to overcome patient, social and systemic level barriers to enable effective communication about HBC.

Perceived challenges to effective communication about HBC with patients were related to the low socio-economic backgrounds of patients attending public hospitals. Such patients were generally believed to have limited education/health literacy, hence limited knowledge of BC, inheritance or family history of cancer, and misconceptions about the cause of the condition. These findings highlight the need for developing accessible information resources, using plain language, to support communication with patients about HBC and its implications for relatives. Whilst resources have been developed for use in HICs, research is needed to co-develop similar resources locally with HCPs and BC patients with low literacy and for use in a context with limited access to genetic testing (van der Giessen et al. 2021; Ahmed et al. 2022). To further enable patient access, such information resources should be developed in written, audio, and video formats.

The main systemic barrier to communication about HBC with patients attending public hospitals was perceived by HCPs as the lack of genetic testing. HCPs recognised patients may be at risk for HBC based on their age of diagnosis or family history, but they had concerns about the extent to which BC in such cases was due to genetic versus environmental factors (family lifestyle and/or environment). Therefore, there is a need for genetic testing, so that HCPs may communicate with patients about HBC based on clinical evidence for the condition. Genetic testing is further justified by studies showing that BRCA mutations account for a substantial proportion of HBC and early-onset BC cases in Pakistan (Rashid et al. 2006; Abbas et al. 2019; Tariq et al. 2021; Vohra et al. 2022; Rashid et al. 2022), hence encouraging genetic risk assessment for patients diagnosed with BC when aged 45 years or younger and patients with triple-negative BC regardless of age (NICE 2019). Furthermore, patients with early-onset BC may have a sub-optimal response to therapy compared to cancer observed in older women which requires hormonal manipulation (Vohra et al. 2022). In Pakistan, genetic testing is currently available for BRCA 1 and 2 genes for BC patients and their first-degree relatives, but only via private health services. Political intervention is needed to provide equitable access to genetic testing for all patients, particularly socioeconomically disadvantaged individuals, to enable improved patient outcomes through targeted therapy and to enable communication of HBC risks for relatives.

The provision of genetic testing in public hospitals may not be an option in Pakistan or other resource-limited LMICs in the immediate future. Therefore, there is a need for government policies, clinical guidance documents (similar to those in HICs) (NICE 2019), and assessment tools to identify patients with a family history of BC and manage those at risk for HBC accordingly. Clinical guidelines are also needed on the extent to which HCPs should use other “genetic information”, such as histological analysis of tumours that strongly suggest hereditary cancer, information from BC risk prediction models suggestive of HBC, and patients’ family history of BC (Black et al. 2013). The application of a broader definition of genetic information could support HCPs to provide HBC information to patients in the absence of genetic testing.

HCPs considered communication with patients’ relatives about HBC beyond their remit. This finding aligns with research emphasising that patients are ultimately responsible for intrafamilial communication (Young et al. 2020). Nevertheless, HCPs acknowledged the challenges for patients in initiating such communication. Similarly, research shows that patients need to understand complicated genetic information themselves, then communicate this sensitive information to relatives with varying levels of education and from different generations, in addition to navigating family dynamics (Black et al. 2013), in a cultural context where there is social stigma and shame related to having BC. Research also shows that despite recognition of the benefits of genetic information, patients find it practically and morally challenging to initiate these conversations with family members, for example, they are reluctant to cause fear and stress in relatives, and concerned about the impact of genetic information on family relationships (Black et al. 2013; Lieberman et al. 2018). Therefore, similar to others (Saeed et al. 2021), HCPs advocated the need for interventions to reduce barriers to diagnosis and treatment of BC by raising awareness about the condition and developing resources to support intrafamilial communication, including written information for patients to pass on to relatives, and the availability of genetic counselling. In addition, research should explore the implications of introducing genetic counsellors in cancer clinics and whether this could help support intrafamilial communication.

The need to reduce BC-related stigma and promote screening for the condition via public awareness campaigns is well acknowledged (Dey et al. 2016). In Pakistan, limited resources have resulted in sporadic, local, and short-duration awareness campaigns. Research is needed to co-develop national or at least provincial awareness campaigns, particularly for people with varying levels of health literacy and for teenagers and adolescents. Research is also needed on the most effective ways of delivering BC awareness campaigns, particularly in rural areas, and the extent to which such sensitive information is best communicated via community or religious leaders versus HCPs.

Genetic counselling can also play an important role in facilitating intrafamilial communication, providing crucial support for HCPs with limited time. However, HCPs in Pakistan have little training on genetic counselling. Unlike HICs, genetic counselling is not a part of the healthcare system in Pakistan. Nevertheless, genetic counselling is available via the Government-funded “Punjab Thalassaemia Prevention Programme” (https://ptgd.punjab.gov.pk/genetic_counselling), albeit for thalassaemia only. The sustained and successful achievements of this organisation in the prevention of thalassaemia for over a decade have led to further funding from the Government of Punjab, which now includes the prevention of other genetic conditions. This enhanced service will be known as the Punjab Thalassaemia and Other Genetic Disorder Prevention and Research Institute. Therefore, it is timely for policymakers and HCPs to consider how this organisation could include genetic testing and counselling services for HBC.

HCPs in our study had different views about the extent to which HBC was an issue. The prevalence of BC and HBC is not known in Pakistan because of the lack of national BC registries and epidemiological studies, so there is little evidence on the extent to which HBC is an issue in Pakistan. To inform policy and the development of genetic services for HBC, epidemiological research is needed on this in Pakistan. Such research could better enable HCPs to recognise the need for informing patients about HBC and intrafamilial communication with at-risk relatives.

This study presents an in-depth insight of HCPs’ perceptions of communication with patients and their families about HBC in a low–middle-income country. The study is limited to HPCs in a public sector hospital in the Punjab province. Further research is needed with HCPs working in the private sector and in other provinces. Also, the study includes mainly female HCPs. This is because few males enter the field of breast cancer in Pakistan.

In conclusion, our findings suggest HCPs would value the development of interventions to support communication between HCPs and patients and intrafamilial communication between patients and relatives about HBC. Much research and political support are needed to address patient, social, and systemic-level barriers to facilitate HCPs’ communication about HBC.

## Data Availability

The datasets generated and/or analysed during the current study are available from the corresponding author on reasonable request.
